# Using online methods to recruit participants into mental health clinical trials: considerations and recommendations from the RE-MIND study

**DOI:** 10.1186/s13063-024-08435-9

**Published:** 2024-09-07

**Authors:** Mais Iflaifel, Charlotte L. Hall, Heidi R. Green, Andrew Willis, Stefan Rennick-Egglestone, Edmund Juszczak, Mark Townsend, Jennifer Martin, Kirsty Sprange

**Affiliations:** 1 Nottingham Clinical Trials Unit, University of Nottingham, Nottingham, UK; 2 Institute of Mental Health, School of Medicine, NIHR MindTech MedTech HRC, Mental Health and Clinical Neurosciences, University of Nottingham, Innovation Park, Triumph Road, Nottingham, UK; 3 Institute of Mental Health, NIHR Nottingham Biomedical Research Centre, University of Nottingham, Innovation Park, Triumph Road, Nottingham, UK; 4 Health Services Research Unit, University of Aberdeen, Aberdeen, UK; 5 COUCH Health, Manchester, UK; 6 Leicester/Diabetes Research Centre, Centre for Ethnic Health Research, University of Leicester, Leicester, UK; 7 School of Health Sciences, Institute of Mental Health, University of Nottingham, Nottingham, UK; 8 NIHR Evaluation, Trials and Studies Coordinating Centre (NETSCC), Southampton, UK

**Keywords:** Mental health, Clinical trial, Recruitment, Recommendations, Diversity, Inclusivity

## Abstract

**Background:**

Ensuring diversity in clinical trials can be a challenge, which may be exacerbated when recruiting vulnerable populations, such as participants with mental health illness. As recruitment continues to be the major cause of trial delays, researchers are turning to online recruitment strategies, e.g. social media, to reach a wider population and reduce recruitment time and costs. There is mixed evidence for the use of online recruitment strategies; therefore, the REcruitment in Mental health trials: broadening the ‘net’, opportunities for INclusivity through online methoDs (RE-MIND) study aimed to identify evidence and provide guidance for use of online strategies in recruitment to mental health trials, with a focus on whether online strategies can enhance inclusivity. This commentary, as part of the RE-MIND study, focusses on providing recommendations for recruitment strategy selection in future research with the aim to improve trial efficiency.

A mixed-methods approach was employed involving three work packages: (I) an evidence review of a cohort of 97 recently published randomised controlled trials/feasibility or pilot studies in mental health to assess the impact of online versus offline recruitment; (II) a qualitative study investigating the experiences of *n* = 23 key stakeholders on use of an online recruitment approach in mental health clinical trials; (III) combining the results of WP1 and WP2 to produce recommendations on the use of an online recruitment strategy in mental health clinical trials. The findings from WP1 and 2 have been published elsewhere; this commentary represents the results of the third work package.

**Conclusion:**

For external validity, clinical trial participants should reflect the populations that will ultimately receive the interventions being tested, if proven effective. To guide researchers on their options for inclusive recruitment strategies, we have developed a list of considerations and practical recommendations on how to maximise the use of online recruitment methods.

## Introduction

Recruitment to clinical trials is challenging, and those in mental health research are no exception and people with mental health illness have been identified as an under-served group in health research [1]. The importance of broader representation of under-served populations in clinical trials is already well established to ensure they reflect the populations that stand to benefit from the intervention being tested [2]. The question is how do we improve recruitment when we already know that mental health service use is proportionately lower for the socioeconomically disadvantaged [3], males [4], people from ethnic minority backgrounds [5], and older participants or those living in more rural areas [6]. Traditionally, recruitment into mental health trials has been dependent upon face-to-face referrals and therefore limited to those individuals actively seeking service intervention, thus perpetuating the problem [7]. Furthermore, increasing pressures on mental health services has become an obstacle to the delivery of trials using this approach; however, technological advances are allowing researchers to be more creative and dynamic in their choice of recruitment strategies to target potential participants typically outside of services and reach wider groups of people [8]. Despite this potential, deciding upon what might be the best recruitment strategy for those living with mental health illness needs further careful consideration.

To help address, this we conducted a study, “REcruitment in Mental health trials: broadening the ‘net’, opportunities for Inclusivity through online methoDs’ (RE-MIND)” https://www.nctu.ac.uk/our-research/methodology.aspx. The objective was to explore the use of offline and online recruitment strategies with the aim of helping researchers improve recruitment reach and increase the efficiency of clinical trials of mental health interventions.

This project focussed on the recruitment strategy used to make the initial approach to potential participants, informing them about an active clinical trial. As our focus was on the initial stage in recruitment, we did not cover issues surrounding the consent process itself. Despite this, we acknowledge the importance of the methods of taking informed consent, and this should be considered when deciding on a recruitment strategy.

The RE-MIND study consisted of two work packages which have been published separately [9, 10]. First is an evidence review of 97 recently published randomised controlled trials (RCTs) and randomised feasibility/pilot studies in mental health to assess the impact of online recruitment versus offline recruitment in clinical trials [9]. Second is a qualitative study investigating the experiences, opinions, and ideas of *n* = 23 key stakeholders (research staff and patients and public involvement members with experience working in mental health research) on the use of online recruitment as an approach in mental health clinical trials [10]. The findings were then triangulated [11] by researchers MI, KS, and CLH to develop draft considerations and practical recommendations which then underwent a review process by the study Advisory Group (HRG, AW, SRE, EJ, MT, and JM) who have experience in digital research, design, and delivery of online and offline RCTs and equality, diversity, and inclusion resulting in the final recommendations.

Throughout the RE-MIND study, we used the following definitions to broadly categorise offline or online recruitment. These definitions describe an overarching strategy to recruitment:

- *Online recruitment strategies*—the use of Internet technologies such as social media advertisements, Google search engine advertisements, and other website campaigns [12].
- *Offline recruitment strategies*—in-clinic recruitment, approaching potential participants through mail and telephone using health records and registers, media campaigns, newspaper advertisements, and input during radio and television interviews [12].

In this commentary, we present a list of considerations and practical recommendations for research teams on the use of online recruitment of participants into mental health clinical trials with the aim to improve recruitment efficiency in clinical trials of mental health interventions. It is worth noting that although the RE-MIND study focussed on mental health interventions the findings may also be beneficial in wider clinical research.

## Recommendations

### Complexity of mental illness

Severity of mental health illness has previously been identified as a barrier to participation in mental health research [13, 14]. RE-MIND reported that the type of mental health illness, its stage, participants’ feelings about their illness, and carers’ responsibilities were key factors when selecting a recruitment strategy [10]. Alongside meaningful and authentic patient and public involvement (PPI) to guide and inform the recruitment strategy, using a multi-method approach to recruitment could improve accessibility and inclusivity, by supporting the diverse and changing needs of those living with mental health illness.

#### Considerations

I. Consider any relationships between recruitment strategy and mental health symptomatology:
  For example, individuals with learning disabilities, autism, anxiety, or obsessive compulsive disorder may have difficulty interfacing in public and therefore may benefit from online recruitment.
  Online recruitment may, however, be a barrier for other mental health illnesses such as low mood disorders, depression, personality disorders, and psychosis where an in-person approach offers more security, contact, and support to the individual.
II. Consider using the stage or severity of illness to inform recruitment method:
  Will the person’s diagnostic and treatment experience impact on selection of recruitment method, for example, both patients and carers may be reluctant to talk about or need more time to process a diagnosis in the early stages?
  Are personal cues, such as body language, important for communicating with your participants and supporting greater engagement in a trial, for example, recognising changes in mental health state, physical discomfort, increasing tics, loss of concentration, fatigue, etc.?
  Is personal contact preferable or more encouraging, for example, for building rapport and trust with the individual?
III. Consider whether the recruitment method selected may impact on any experience of stigma around mental health:
  Providing a virtual safe space (online) may be beneficial, but the safety of this space relies on participants having secure and private access to a safe space and a device that can access the Internet.
IV. Consider the impact of the relationship between participants living with mental illness and the research team:
  Trusting relationships are deemed important for both recruitment and retention of participants living with mental ill health. Knowing that a health care provider understands an illness and can offer personalised support can be reassuring.
  Will your recruitment strategy choices contribute to maintaining or building trust with this group? Online recruitment, such as social media, can be seen as distant and disengaging compared to in-person recruitment. Regular trial updates and information sharing through short videos or live ‘chats’ may help ‘humanise’ the trial on digital platforms.

#### Recommendations

1.1 Develop your recruitment approach (offline/online/mixed) by working in partnership with potential participants and members of the public that share characteristics with your target population group. You can identify PPI contributors through your local employing organisation or through professional or existing research or public contributor groups such as Sprouting Minds https://digitalyouth.ac.uk/the-digital-youth-programme/about-sprouting-minds/. Please note that most UK National Health Service (NHS) Trusts have established PPI Groups.
1.2 Build in flexibility where possible at the protocol development stage, to ensure that participants with fluctuating symptoms can remain engaged in a safe and supported way. This may be achieved in several ways, for example, by offering a mixed recruitment strategy to allow individuals to choose how they want to participate. Alternatively, you may select an online recruitment strategy via Facebook for the initial approach to participate but then build in telephone or in-person opportunities for eligibility checks or follow-ups. It is important to ensure participants know that these options exist at the earliest opportunity.

### Inclusivity

RE-MIND identified a number of specific challenges to inclusive recruitment into mental health clinical trials. Continuing stigma surrounding mental health was a significant factor on a political, cultural, community, and individual level, underpinned by lack of education and mistrust of services and research [10]. In addition, lack of researcher skills and experience in inclusive recruitment strategies has also been found to contribute to underrepresentation in clinical research [15, 16]. This highlights the critical role of PPI in understanding a trial population’s needs. It is also vital to educate researchers on equality and diversity, to enable co-design and selection of suitable recruitment methods to improve representation in mental health clinical trials, for example, through better implementation of the UK’s National Institute for Health Research (NIHR) INCLUDE ethnicity framework [1].

#### Considerations

I. Consider the impact of the relationship between participants from marginalised groups and the research team:
  Will your recruitment strategy choices contribute to maintaining or building trust with this group, for example, those living with mental health illness in rural or under-served communities may benefit from an online recruitment strategy?
  Can you develop relationships with local and/or national community groups to build trust in your research? Identify community group leaders who will advocate for your research.
  Do you have connections with trusted members of the community to support the building and development of relationships to facilitate inclusive recruitment? This can be in-person or online for example. through administrators of Facebook groups, libraries, or leaders of interest groups.
  Do you have a PPI member with lived experience on your research team who can advocate for your research with community groups? Establishing connections through shared experience can help break down barriers of mistrust and misunderstanding.
II. Consider which recruitment methods your target participant populations may prefer. Living with a mental health illness can be complex due to fluctuating health status or exacerbation of symptoms:
  Think about what factors may be most important to them, e.g. if they are working, parents, carers, and/or attending school, then convenience may be the main factor to target.
  Consider the range of media platforms available to target people who are educationally or socioeconomically diverse.
  If local IT access, e.g. poor Internet access is known in a geographical area, consider using mixed methods for recruitment to improve inclusivity.
III. Consider information provision and accessibility when selecting your recruitment methods:
  Consider whether the methods you are using to recruit and retain participants allow for language (written and/or spoken) needs to be met, e.g., using a translation service.
  Consider whether the recruitment method selected allows you to adequately communicate what you need to your participants for example:
    Social media platforms such as X (previously Twitter) or use of SMS text-based services have character limitations. Could any language or phrasing lead to misinterpretation or misunderstanding
    Use of clinical or diagnostic terms, phrases and labels when considering issues of stigma.
  If you are using offline methods, are they accessible for people in a physical sense? E.g., people with motor/mobility needs, or visual or auditory difficulties.
  If you are using online methods, are the colours, font, and imagery that you are using inclusive? E.g., alt text for images, colour blindness, colour contrast and font readability.

#### Recommendations

2.1 Work in partnership with people with lived experience and members of the public that share characteristics with your target population group. Explore the needs of both the trial team and the target population group and select methods that are effective for both parties.
2.2 Greater sensitivities and confidentiality in mental health care mean that relationships and trust are critical, which may be easier to facilitate face-to-face. However, online recruitment may offer greater flexibility and convenience for participants, for example, by supporting those who may find in-person contact challenging due to their illness. When selecting a recruitment strategy , be mindful of both advantages and drawbacks of the strategy used.
2.3 Avoid stereotypes, particularly related to age, when thinking about online methods. For example, technology as a barrier is likely reduced with each generation as well as recent necessity to engage with digital communications (e.g. smartphones, WhatsApp, Facebook, videoconferencing platforms) due to the COVID pandemic.
2.4 Identify the main demographic characteristic(s) that is important to engage with your trial, and then consider how other characteristics may impact how they react to the recruitment strategy you have in mind. For example, if you know you want to include young people, consider using TikTok, whereas Facebook may be preferable for older participants. It is important to think about other characteristics that may impact if/how social media is used, e.g. mental health status, socioeconomic status, health status, gender.
2.5 There are a growing number of community-led mental illness specific support groups on social media. Can you access and/or engage these groups to help with recruitment? Care should be taken not to harm the safe spaces afforded by these groups, for example for a researcher joining a group purely for the purpose of trial promotion.

### Data management

RE-MIND found that for people living with mental health illness, there remained a significant element of fear and mistrust in using online methods underpinned by the stigma and vulnerability of mental health illness with the potential for confidentiality to be broken [10]. Understanding safeguards for the range of digital platforms was particularly complex and in line with other research suggests better regulation is needed of digital platforms [17], which at times were not deemed as stringent as clinical trial requirements.

#### Considerations

I. Consider putting appropriate safeguards in place for the recruitment methods selected, e.g. firewalls, General data Protection Regulation (GDPR), secure server.
  Can you use a quick response (QR) code to improve security and safety. A QR code is an image scannable by a digital device that can impart information.
  Does your organisation have data management policies for use of digital platforms such as social media that must be adhered to? Consider local policies required for multi-site trials.
  Is your recruitment method a credible source, e.g. not mistaken for spam, phishing?
  Allocate a moderator for engagement with online public groups to ensure safeguarding and wellbeing of people engaging with the content.
  How will you inform potential participants about how their data will be shared and/or managed online?
II. Consider the resources required to adequately manage large numbers of enquiries generated by online strategies:
  Do you have the resources to support the additional work associated with screening and monitoring of data quality?
  Ensure that eligibility criteria are clearly communicated to potential participants.

#### Recommendations

3.1 Invest adequate time and resources in ensuring your data management systems are secure and safe for participants. You may want to make use restrictive software features for online methods.
3.2 Invest time to ensure security and safety methods are communicated clearly. You should work in partnership with potential participants and members of the public that share characteristics with your potential participant group to do this.

### Staff training and support

The process of targeting recruitment using an online strategy has been considered as more time-efficient and cost-effective than traditional offline (in-person) recruitment [12]. However, knowledge of digital platforms and access to organisational and technical support and funding were the most common challenges researchers cited when selecting a recruitment strategy in the RE-MIND study [10]. It appears that despite advances in technology offering greater opportunity to reach wider audiences, many of these advances remain underutilised without adequate support and resources.

#### Considerations

I. Consider identifying trials involving similar participant populations to learn from their experience of recruitment:
  For example, information on trials can be accessed from ClinicalTrials.Gov, PubMed, etc.
  Remember that this relies on adequate reporting of recruitment strategy.
  Think about how previous trials could have been improved.
II. Consider the impact of researchers/recruiters being in/adequately trained and knowledgeable on how to use the online recruitment methods you have chosen:
  If you are using social media, does your organisation have policies and/or expertise that can be used to support engagement on specific platforms
  Does your organisation have procedures for payment for social media promotion?
  Do you have local services available to support at an organisational level when things go wrong, e.g. IT, marketing, or communications teams?
  Ensure your recruitment methods are appropriately funded, for example, advertising costs per click; do you need a professional designer to produce visual summaries of the research, such as infographics?
  Do you have lived experience patient and public input on the selection of recruitment strategy, including content and presentation?

#### Recommendations

4.1 Make a conscious effort to learn from previous trials aimed at the populations you are intending to recruit. Reflect upon how these trials may differ from yours and how that may impact your selection of recruitment process (e.g., severity or stage of mental health illness, intervention type, locality, country, setting, healthcare system, culture).
4.2 Ensure research teams are adequately trained on systems and software, and that they know where to go when systems fail, or if they have unanswered questions.

## Conclusions

This list of considerations and recommendations is based on the experiences of key partners and the findings from the RE-MIND project, outlining factors to consider when planning recruitment strategies in mental health research/clinical trials. It should be used as a starting point for discussions among the trial team. We acknowledge the potential limitations of each consideration in context of individual and/or organisational capacity, funding and resources available.

The process of selecting a suitable recruitment method should give due consideration to the study population as well as the resources (including staff time and training) needed to implement that method. The ideal juncture to do this is when writing a trial grant funding proposal to ensure adequate resourcing. However, we encourage trial teams that are struggling to recruit to use our considerations and recommendations to re-evaluate their approach to recruitment.

The considerations are designed to be used flexibly based on the target population to be recruited. Greater consideration should be given to using online or mixed methods recruitment strategies that adopt a tailored approach, offering flexibility and choice, to enable wider participation. For future work, we recommend revisiting and re-evaluating these considerations after they have been implemented in practical settings. This process of reassessment will allow us to gain valuable insights into the real-world impact and effectiveness of our proposed strategies. It will also enable us to make necessary adjustments, fine-tune our recommendations, and ensure their continued relevance and success in evolving contexts.

## Data Availability

The data collected, used, and/or analysed during the current study are available from the Nottingham Clinical Trials Unit (NCTU) via the corresponding author on reasonable request.

## Abbreviations

GDPR: General Data Protection Regulation
PPI: Patient and public involvement
NCTU: Nottingham Clinical Trials Unit
NHS: National health Service
NIHR: National Institute for Health and Care Research
QR: Quick response
RCT: Randomised controlled trial
SMS: Short message service
RE-MIND: REcruitment in Mental health trials: broadening the ‘net’, opportunities for INclusivity through online methoDs
UK: United Kingdom
